# An internal health systems research portfolio assessment of a low-income country research institution

**DOI:** 10.1186/1478-4505-8-8

**Published:** 2010-04-06

**Authors:** Tracey P Koehlmoos, Damian G Walker, Rukshana Gazi

**Affiliations:** 1 Health and Family Planning Systems Programme, ICDDR,B, Mohakhali, Dhaka 1212, Bangladesh; 2 Health Systems Program, Department of International Health, Bloomberg School of Public Health, Johns Hopkins University, 615 N. Wolfe Street, Baltimore MD 21205, USA; 3 Health Systems and Economics Unit, Health Systems and Infectious Diseases Division, ICDDR,B, Mohakhali, Dhaka 1212, Bangladesh

## Abstract

**Background:**

In order to determine the type and amount of health systems research being conducted within ICDDR,B (also known as the Centre), a leading research institution in Bangladesh, an internal review of all on-going research protocols was conducted in September 2007.

**Methods:**

A review of all ongoing research protocols within the Centre was conducted. The names of the investigators and the institutional divisions of the protocols were removed in order to decrease the amount of reviewer bias. The building blocks of the World Health Organization's "Framework for Action" on health systems was used to categorize the protocols considered to be health systems research projects. Several additional items were collected, e.g. the highest level of education completed by the Principal Investigator. A total dollar value was placed on the health systems research portfolio of the institution based on the budgets of the selected protocols.

**Results:**

As of September 2007 16 out of 118 (13.5%) reviewed protocols were considered to be health systems research projects. Results of the six building blocks of the health system categorization demonstrated that a majority of these protocols involved elements of health services delivery. There was very little engagement in more downstream systems and policy research that involved leadership and governance of the health system. Eleven of the HSR studies were local in scope, while there was only one study that has a multinational focus. The Centre's total dollar value for the health systems research project portfolio added up to US$ 3,723,331.

**Conclusions:**

This internal review can serve as a snap shot of on-going activities, and as a baseline for future assessments against which to monitor progress in the area of health systems research. Further, it can serve as a model for other institutions striving to assess and develop health systems research programmes and capacity.

## Background

In recent years, heath systems research has emerged as an increasingly important aspect in the progress of nations toward achieving the Millennium Development Goals [1]. Accordingly, there has been a significant increase in health systems research and health systems strengthening funding opportunities, examples of which include the 2007 funding round of the AusAID Development Research Awards and Round 8 of the Global Fund to Fight AIDS, Tuberculosis and Malaria.

While there is little doubt as to the importance of health systems research and the need to strengthen health systems to improve outcomes, globally and locally there is misunderstanding as to the scope and activity of health systems research [2]. One example of local misunderstanding is a senior scientist at ICDDR,B (also known as the Centre) conducting a randomized controlled trial of a locally available unripe fruit for the treatment of diarrheal disease, and considering it to be health systems research because the research concerned a health issue and ultimately the findings of the RCT might be adopted by service providers in the health system although research activities in support of that goal were not part of the RCT. Conversely, scientists engaged in health systems research may not know that they are conducting such research leading to a failure to properly translate the findings into action by not recognizing the weaknesses in the health system and how they would impact any potential change to the health sector. It is possible that many researchers based in low- and middle-income countries share this lack of understanding. Thus, it is important to establish a common understanding of what exactly health systems research is and entails.

As early as 1991 Varkevisser, Pathmanathan, and Brownlee (1991) defined health systems research in low-income settings as work which is "ultimately concerned with improving the health of people and communities by enhancing the efficiency and effectiveness of the health system as an integral part of the overall process of socio-economic development" [3]. However, more recently, the World Health Organization (WHO) definition of a health system more broadly includes "all activities whose purpose it is to restore and maintain health" which lends itself to the definition of health systems research as being those investigations that seek to evaluate or promote coverage, quality, efficacy and efficiency within the health system [2,4]. The WHO further developed a Framework for Action based upon six building blocks of a health system: Service Delivery, Information and Evidence, Medical Products and Technologies, Health Workforce, Health Financing and Leadership and Governance [4]. In addition, health systems and policy research should be considered downstream, and should look at "policies, organizations and programs, but does not address clinical management of patients or basic scientific research" [2]. The definition of Health Systems Research used in this evaluation is based on the later definitions of the WHO.

**Figure 1:**
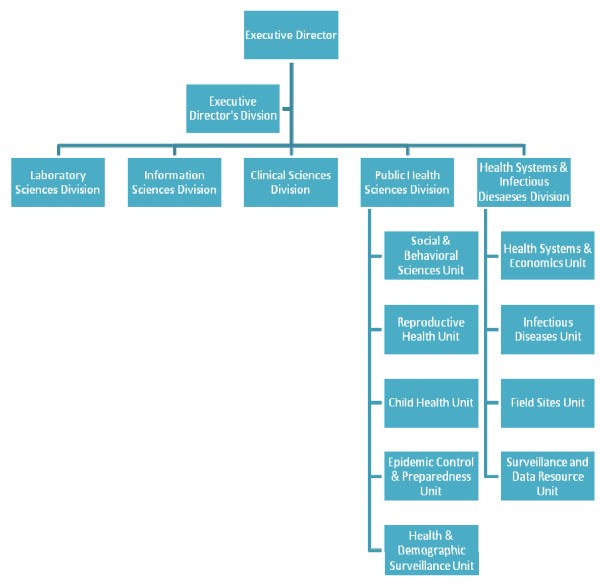
**ICDDR,B Structure, detailing PHSD & HSID**.

This paper discusses the experience of an international research institution in a low-income country as it endeavors to meet the needs both of its host nation and to stay current in the field of health systems research. The Centre began its existence as a specialty infectious disease research hospital but over the last 50 years it has expanded to become a population and health research centre with two hospitals, eight field sites and more than 2,500 employees of whom around 170 are research scientists. The Centre has five divisions: Laboratory Sciences, Clinical Sciences, Public Health Sciences, and Health Systems & Infectious Diseases, Information Sciences and an Executive Director's Division. Areas of specialty covered by the Centre include demographic surveillance, infectious disease, diarrheal disease research, HIV/AIDS, nutrition, maternal and child health research. Over a two year period, the Health Systems and Infectious Diseases Division (HSID) (2006) and the Public Health Sciences Division (PHSD) (2007) underwent reviews by external experts. Figure 1 contains an organogram of the Centre including unit level details of HSID and PHSD. In both cases despite having a health systems division, a health systems and economics unit and being engaged in international collaborations like the Future Health Systems Consortium [5,6], the evaluators determined that there was neither a clear picture of health systems research activity within the Centre nor a clear understanding among scientists of the comprehensive framework that is health systems research.

The Centre's core health systems capacity resides within the Health Systems and Economics Unit of HSID and the Social and Behavioral Sciences Unit of PHSD. Until June 2006 the Health Systems and Economics Unit had been funded primarily as a single-donor programme that conducted operational research to improve the service delivery system of the public health system in Bangladesh. In addition a number of researchers beyond these groups conduct health systems research as part of the nutrition and child health programmes. The latter group of scientists is excluded from the following description of the Centre's critical mass of health systems scientists because they would not identify themselves as health systems researchers and did not categorize their work as health systems research. Within the Health Systems and Economics Unit and the Social and Behavior Sciences Unit there are a total of 25 scientists ranging from those just out of a masters programme to those with 20+ years of experience. Nine have PhDs. There are two full-time and one visiting scientists who have PhDs and are international hires. The following disciplines are represented: medical anthropology, demography, health administration and management, medicine, nutrition, economics, public health, epidemiology and health services research. An overview of the Centre is provided below:

ICDDR,B is an international health research institution located in Dhaka, the capital of Bangladesh. In collaboration with partners from academic and research institutions throughout the world, the Centre conducts research, training and extension activities as well as programme-based activities. The Centre's research work led to the development of ORS, which is used worldwide and is estimated to save three million lives annually. In May 2001, the Centre became the first recipient of a Gates Award for Global Health in recognition of its outstanding achievements.

The Centre is home to highly qualified national and international scientists and professional staff. It has a cross-cultural environment with 95% local staff that includes researchers, medical officers, administrators, and health workers, and 5% international staff primarily from academic and research institutions engaged in global health research. A mix of various health professionals including public health scientists, laboratory scientists, clinicians, nutritionists, epidemiologists, demographers, health economists, social and behavioural scientists, IT professionals, experts in emerging and re-emerging infectious diseases, vaccine scientists, and others are working together with a broad aim of finding solutions for global health problems.

The Centre serves as a model of collaboration with the government and people of Bangladesh in a way that respects and supports each other to the ultimate benefit of the health of the nation and of other countries in the region. The longitudinal data collection systems at the Centre's rural and urban demographically defined field sites are unique and serve as a model for the formation of demographic systems elsewhere in the world.

A 2003 survey of health policy and systems research (HPSR) institutions found that on average HPSR producers in low- and middle-income countries were mostly small public units or institutions with eight researchers. The average HPSR portfolio contained three projects and a project portfolio valued at US$ 155,226 in 2003 [7]. The authors of the survey concluded that it may be useful for these institutions to conceptualize and develop a health systems research portfolio through which current commitment and priorities could be evaluated [8].

In order to determine the type and amount of health systems research being conducted within the Centre, an internal review of all on-going research protocols was conducted. This internal review can serve as a snap shot of on-going activities, serve as a base-line against which to monitor future progress in the area of health systems research and further, and can also be incorporated into future health systems research agenda setting exercises and strategic planning.

## Methods

In order to review all of the ongoing research protocols within the Centre, the Research and Project Support Department (RPSD) developed a complete list of protocol abstracts. A protocol abstract contains the hypothesis and objectives of the full protocol. The names of the investigators and the institutional divisions of the protocols were removed in order to decrease the amount of reviewer bias. Also, members of the RPSD did not participate in the evaluation process.

The framework for health systems research outlined in *Briefing Note 1 *from the Alliance for Health Policy and Systems Research (Alliance HPSR) was used to categorize the protocol abstracts [2]. Protocol abstracts were categorized into three groups: Yes HSR, No HSR, and More Information Needed to Determine. Protocol abstracts that fell into the third category were sent back to RPSD with questions about the protocol that the primary investigator would answer in order to provide greater insight into the scope of research. When that information was collected, it was returned to the evaluators for reconsideration and categorization.

Two researchers (TK, DW) evaluated the protocol abstracts for all on-going research activities being conducted within the Centre. One additional researcher (RG) was called in to manage disagreement by group discussion.

Protocols classified as being health systems research projects were further categorized into health systems building block categories and methodologies as applicable. The building blocks were taken from the WHO "Framework for Action" and include: service delivery, information evidence, medical products and technologies, health workforce, heath financing, leadership and governance [2]. Research activities could be classified into more than one building block based on the scope of work.

**Figure 2:**
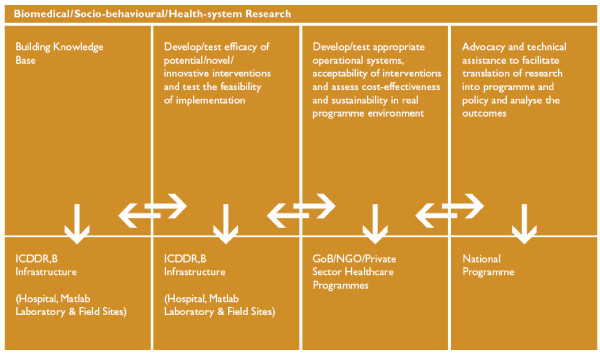
**ICDDR,B Research Model**.

The methodology categories were derived from a conceptual framework of research that exists as a model within the Centre's Strategic Plan [9]. The framework seeks to demonstrate the direction of resources from basic research to efficacy and effectiveness studies and eventually to translation of research results into policy and action [Figure 2]. Thus the categories into which protocols were classified were Building the Knowledge Base, Effectiveness and Efficacy, and Advocacy/Technical Assistance. Further, in order to assess the institution's range of health systems research activities, protocol abstracts judged as HSR were categorized as being local, national or international in scope.

To be consistent with information gathered in a 2003 health systems research capacity analysis conducted by the Alliance for Health Policy and Systems Research, several additional items were collected [7]. Information as to the highest level of education completed by the primary investigator of each health systems research project was compiled. Last, a total dollar value was placed on the health systems research portfolio of the institution based on the selected protocol abstracts.

## Results and Discussion

There were 125 research protocols in progress at the Centre as of September 2007. One hundred and eighteen of these protocols were included in this activity because protocol abstracts could not be produced for the remaining seven protocols. Of the 118 that were dual screened (TK, DW), seven were found to be in dispute. After a brief discussion (led by RG) on each disputed protocol extract, the review team came to a consensus.

The disputed topics are worthy of note because they emerge more from gaps in the knowledge of the protocols than from the actual type of research being conducted within the protocol. For example, one protocol was disputed because of greater knowledge of the study by one of the reviewers. In discussion, it was revealed that researchers at the Centre would conduct the diarrheal disease surveillance activity but that the broader health systems research piece would take place externally so that it was not considered an example of health systems research within the Centre. The second example was of a hand washing education programme, which was excluded as not being part of the health system. A third disputed protocol concerned a Randomized Control Trial of IMCI that seeks to evaluate varying services delivery models while introducing improved management structures accompanied by innovative referral schemes in the intervention group. This dispute was resolved by adding the protocol to the list of health systems research studies taking place at the Centre.

Overall, sixteen out of 118 (13.5%) reviewed protocols were considered to be health systems research projects. The objective of each of the 16 protocols is described in Appendix 1.

Results of the six building blocks of the health system categorization demonstrated that a majority of the health systems research looked at elements of health services delivery. There was very little engagement in more downstream systems and policy research that involved leadership and governance of the health system (see Table 1).

**Table 1:** Characteristics of the protocols

Building blocks of the health system*	Methodology per ICDDR,B Model*	Scope of HSR studies**	Budget of the protocols
Service delivery: 11	Building the Knowledge Base: 8	Local: 11	Less than $50,000: 4
Information and Evidence: 2	Effectiveness/Efficacy: 10	National: 4	$50,000-99,000: 1
Medical Products and Technologies: 4	Advocacy/Technical Assistance: 2	Multinational: 1	$100,000-199,999: 7
Health Workforce: 6			$200,000-499,999: 1
Health Financing: 2			Greater than $500,000: 3
Leadership and Governance: 1			

*Protocols could address >1 category **Protocols limited to one category

Gonzalez-Block and Mills considered the highest level of education in terms of building critical mass [7]. Of the sixteen HSR projects, nine of the primary investigators had Doctorates of Philosophy or equivalent. Fifteen of the projects were lead by someone with at least one Masters degree and one project was lead by a scientists whose highest level of formal education was a Bachelor of Medicine, Bachelor of Surgery (MBBS) but who had worked for the Centre conducting health services research for more than fifteen years. However, four of the project PIs, one of whom was the lead on two projects, would not self-identify as health systems researchers because they worked outside of the two groups described above and work primarily in other disciplines like child health and nutrition.

The Centre's total dollar value for the health systems research project portfolio added up to US$ 3,723,331. This amount is for the duration of the projects rather than the annualized amount during 2007. The total research protocol expenditures of the Centre during 2007 added up to US $9,506,443. The HSR projects ranged in value from a high of US$ 1,000,000 to a low of US$ 34,954 with a mean of US$ 232,708.

There was great variety in the sources of funds for health systems research although the majority of the research was funded by large international donors. Ten of the projects were funded by international donor agencies including the United States Agency for International Development, (USAID) the United Kingdom's Department for International Development (DFID) and the Canadian International Development Agency (CIDA). One study was funded by the Global Fund to Fight AIDS, Tuberculosis and Malaria. One of the projects was funded by the WHO. Two studies were funded by private foundations (the Bill and Melinda Gates Foundation and World Vision). Finally, one project with a dollar value of less than US$ 50,000 was funded by the Centre's own core research funds, which are used to support projects in areas of interest that might generate future research opportunities or present a previously unexplored area. This particular project was looking at health service utilization, health seeking and high risk behavior and needs of the rapidly expanding homeless population in a large urban area.

These results have two limitations. First, they only address on-going protocols as of 18 September 2007. Planned and completed research activities were not taken into consideration by the evaluators. Thus, it is not possible to assess whether there have been changes over time. Second, it was discovered through discussion that some degree of health systems research is taking place outside of the regular channels for registering protocols but in a separate channel that registers work as activities. Examples of health systems research that are registered as activities rather than as protocols included the work of a team that conducts systematic reviews of health systems and policy issues in the non-state sector and the international program headquarters for an initiative to mainstream nutrition into existing maternal, newborn and child health services.

## Conclusions

If it is true that "as demand grows for health policies based on evidence, questions exist as to the capacity of developing countries to produce the HPSR required to meet this challenge" then ICDDR,B must be prepared to invest in capacity building and reorganization in order to meet those needs [8] This paper reviewed the research portfolio of the ICDDR,B, a leading research institution in Bangladesh, a low-income country. Compared to the 176 institutions surveyed by Gonazalez-Block and Mills [7] the Centre had a total project portfolio value twenty-three times higher than the average institution (US$ 3,723,331 v. US$ 155,226) and a portfolio containing five times as many on-going health systems research projects (16 v. 3). While this review has illustrated that the Centre does have some depth in the area of health systems research it was dispersed throughout the various programmes and units of the Centre.

Further, there was pervasive misunderstanding as to the current definition of health systems research and that research done in various fields could indeed be classified as health systems research. Consequently, the Centre should consider the development of training to build understanding and capacity within Bangladesh but also within the Centre itself. A coordinated means of tracking health systems research and policy activity across units within the Centre is needed and consideration should be given to organizational restructuring in order to pool capacity toward obtaining self-identification and consolidating significant critical mass.

Results for the categorical break down into the six building blocks of health systems showed that most of the research took place within the Service Delivery building block (11 out of 16). As in previous portfolio assessment research conducted elsewhere, this study identified the low emphasis given to priority areas such as human resources, policy process, equity, economic policy and health information systems [8]. Thus, the lack of activity in the areas of Leadership and Governance, Information and Evidence, and Health Financing set directions for future avenues of research that could be incorporated into future HPSR research activity. Because of the interconnectedness of the building blocks in the health system, more complex interventions such as scaling-up activities will require the skills to examine the impact on various building blocks [10]. These new directions should be aligned with priorities in Bangladesh, in South Asia and toward achieving the Millennium Development Goals. Further, so that comparisons can be made between the Centre's work, its relationship to national policies and programmes and relevance to global trends, the Centre should consider adopting a more globally accepted model of research categorization such as those described in Donald Stoke's *Pasteur's Quadrant *[11] or Buxton and Hanney's Payback Framework [12].

Moving forward, this internal review can serve as a snap shot of on-going activities. For future assessments it is a base-line against which to monitor progress in the area of health systems research. Furthermore, the assessment can be incorporated into a future health systems research agenda setting exercise, strategic planning and restructuring.

## Competing interests

The authors declare that they have no competing interests.

## Authors' contributions

TK conceived of and designed the study, coordinated the review of the research protocols, conducted the analysis, interpreted the data and drafted the manuscript. RG and DW participated in the review of the research protocols, contributed to the interpretation of the data and helped to draft the manuscript. All authors read and approved the final manuscript.

## Appendix 1

Aim of the HSR protocols

1. To assess the needs of street dwellers and service providers for ESP services and the extent to which those needs are being met through existing facilities

2. To demonstrate the feasibility of distribution of misoprostol by different cadres of health workers, acceptability and effective use of the drug by pregnant women for prevention of PPH, its side-effects and programme effectiveness in reducing post-partum hemorrhage

3. To improve the capacity for client-centered quality reproductive health care and to improve access and utilization through provision of reproductive health commodities and innovative approaches to service delivery

4. To measure the cost-effectiveness of ITNs to reduce the incidence of visceral leishmaniasis compared with no ITNs as well as the economic impact of the disease and the intervention.

5. To document changes in zinc awareness, use and distribution patterns for childhood diarrhea, coinciding with the national scale up of 'Baby Zinc'

6. To explore measures that may facilitate including private sector providers in STI programs

7. To identify gaps between planned and actual performance of the WVB service delivery system in terms of inputs, process and services provided for women and children

8. To evaluate the impact of a package of obstetric and neonatal care that includes community health education, provision of safe delivery, essential newborn care, and management of serious neonatal bacterial infections on neonatal mortality

9. To evaluate two intervention models for improving the management of children with ALRI

10. To provide recommendations for feasible context-specific programming for Safe Motherhood

11. To evaluate the effectiveness of a combination of client- and provider-oriented approaches of partner notification and management in reaching partners of STI clients

12. To assess the quality of services provided by paramedics to abused women

13. To explore the extent and consequences of catastrophic cost for caesarian section delivery.

14. To evaluate the effectiveness of a package of evidence-based interventions through a continuum of care from community to hospital to reduce neonatal, infant, child, and maternal mortality rates

15. To explore policies and regulations regarding nurses, their training, what they are supposed to do as SBAs and what they can actually do, and the community response to nurses as birth attendants and newborn care providers

16. To evaluate the health impact and cost-effectiveness of IMCI, when implemented in the best circumstances and using a randomized experimental design
